# Supplementary material for: High-throughput, image-based phenotyping reveals nutrient-dependent growth facilitation in a grass-legume mixture
Source: PLoS One. 2020 Oct 7;15(10):e0239673. doi: 10.1371/journal.pone.0239673 (PMC7540849; doi:10.1371/journal.pone.0239673)
Supplement: S1 File — (DOCX) [file pone.0239673.s001.docx]

**High-throughput phenotyping reveals nutrient-dependent growth facilitation in grass - legume mixtures**

Ball, K. ^1,3*^, Power, S.A.^1^, Brien, C.^2^, Woodin, S.^3^, Jewell, N.^2^, Berger, B.^2^, Pendall, E.^1^

**SUPPLEMENTARY MATERIALS**

Pearson r^2^ = 0.77, p<0.001

**a**


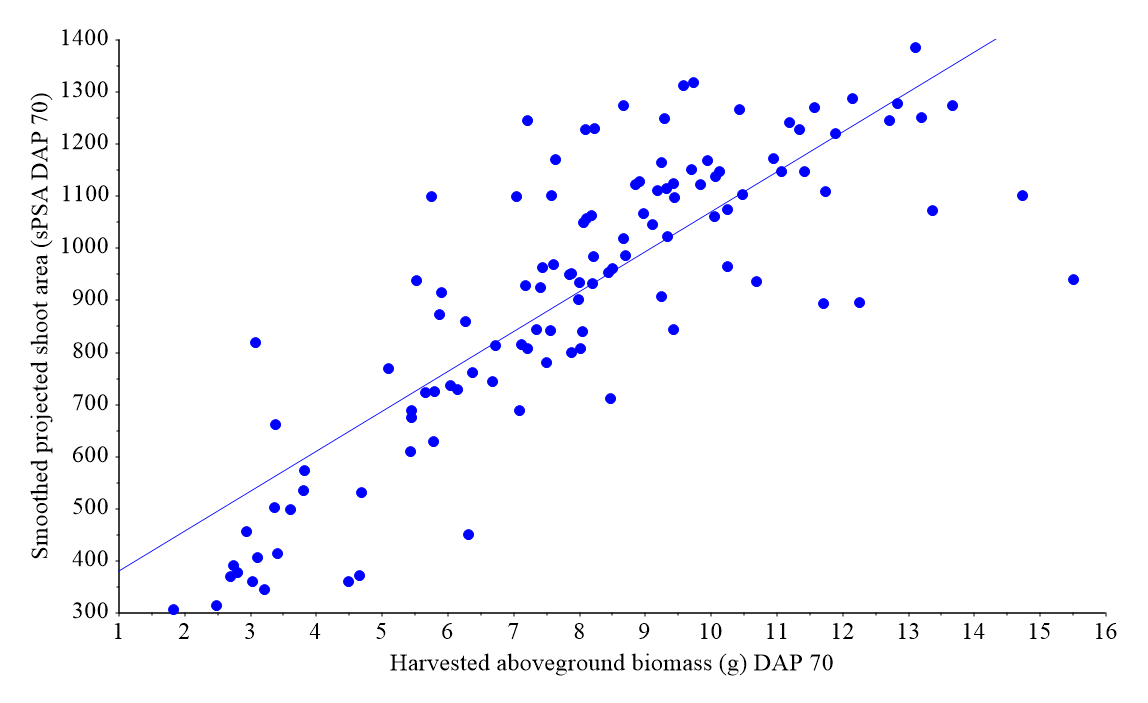


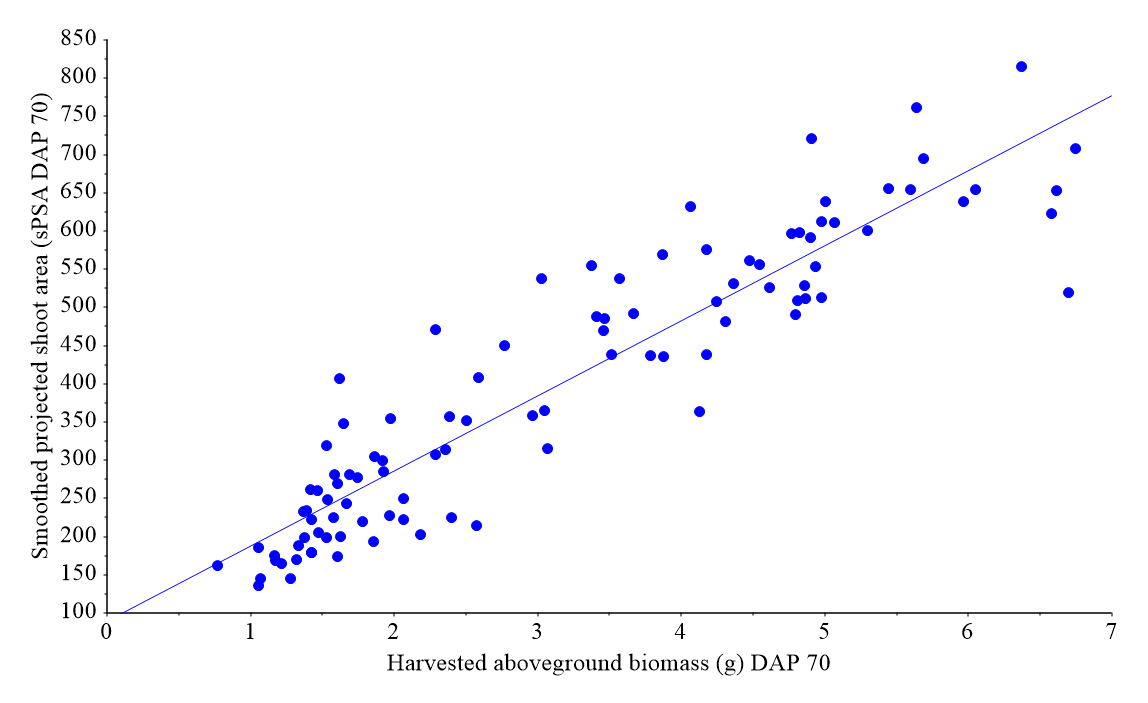


**b**

Pearson r^2^ = 0.85, p<0.001

**
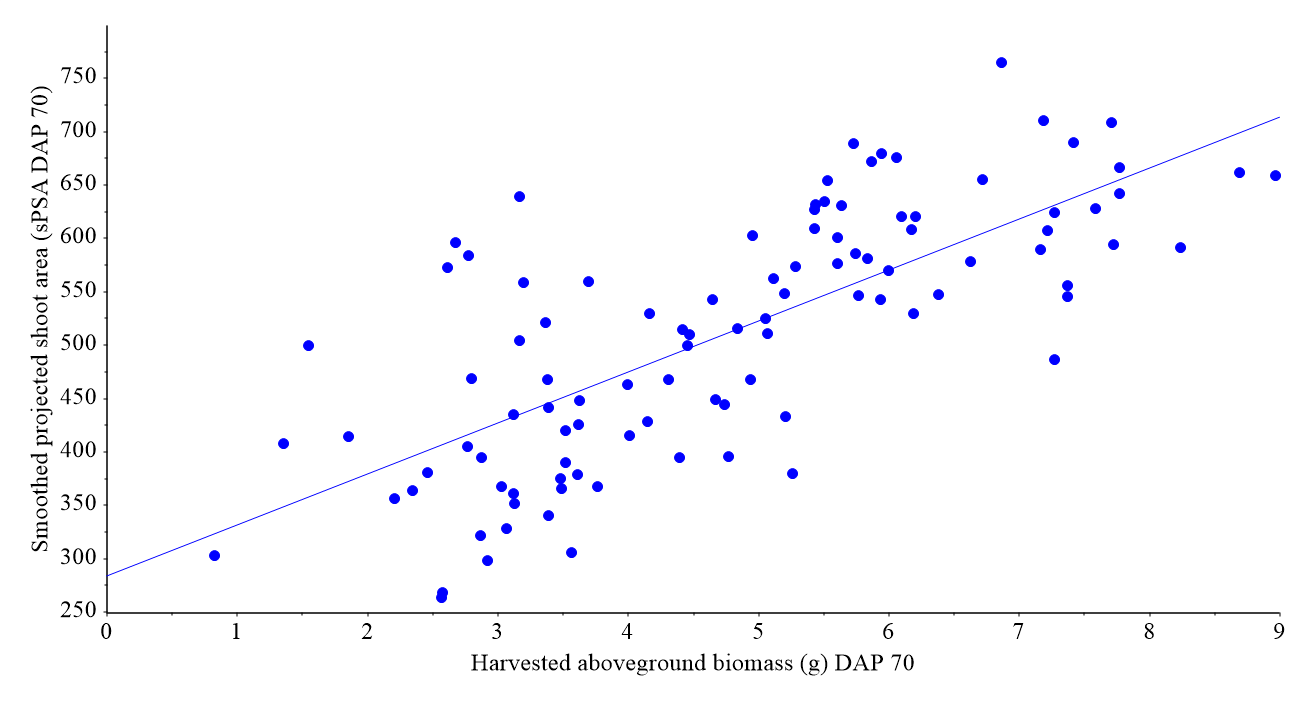
**

**c**

Pearson r^2^ = 0.67, p<0.001

**Figure S1:** sPSA (DAP 70) and harvested biomass correlations for a) total pot (n =119), b) grass only (half pot, n= 238), and c) legume only (n= 240, half pot)

**Plant available nutrients (Extractable N and P)**

Plant available nutrients were measured in plant-free pots using methods described in-text. In the LNLP treatment we measured dry soil plant available N of 0.06 mg g^-1^ [0.05-0.07] and P 0.0071 mg g^-1^ [0.0068-0.0073]. For the HNHP treatment, 0.22 mg g^-1^ [0.21-0.24] of N and (0.0365 mg g^-1^ [0.0309-0.0420]) of P, and for the HNLP treatment 0.18 mg g^-1^ [0.16-0.19] of N and 0.0063 mg g^-1^ [0.0060-0.0066] of P. In the LNHP treatment we measured 0.06 mg g^-1^ [0.05-0.07] of N and 0.0390 mg g^-1^ [0.0366-0.0415] of P.

**
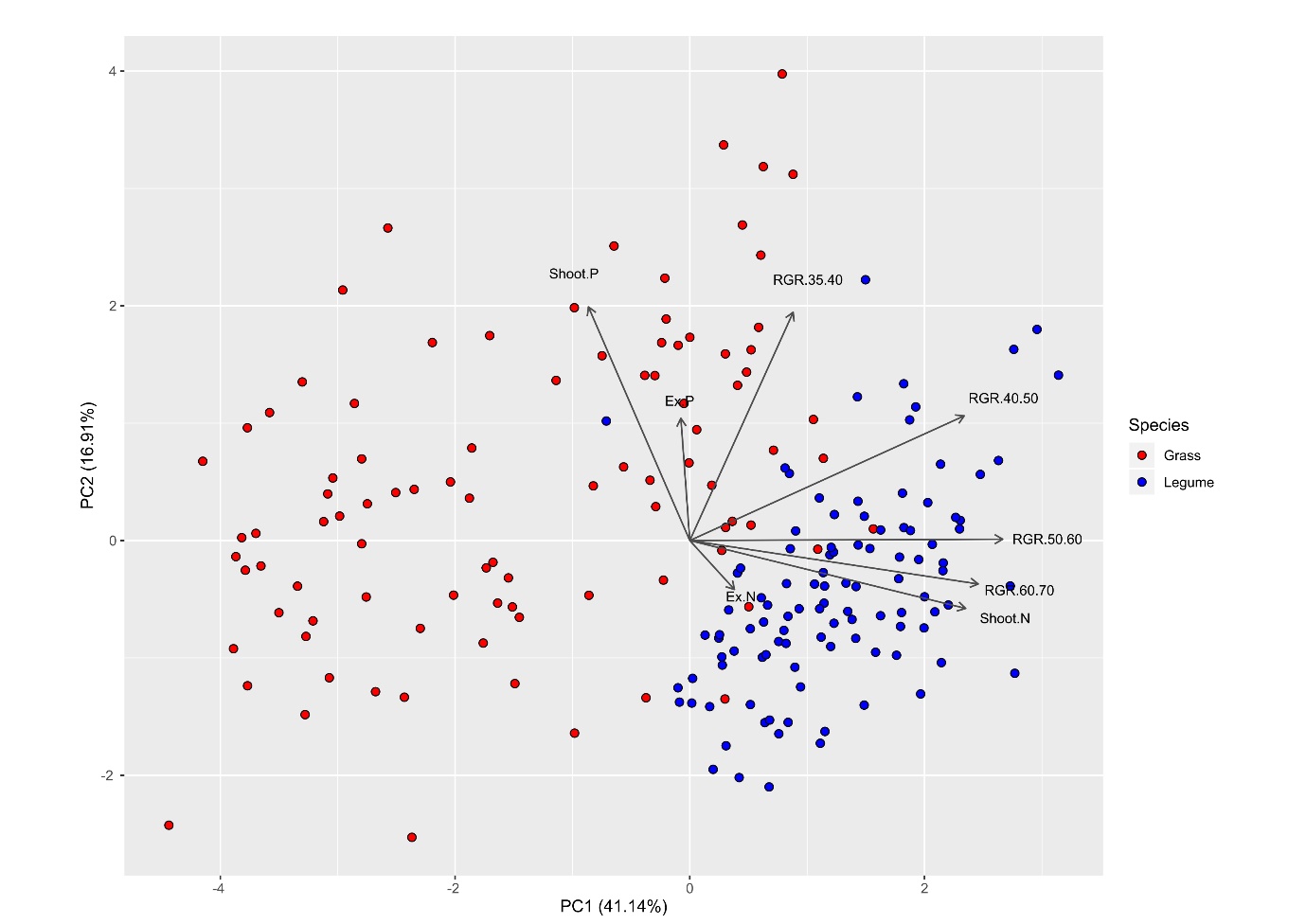
Figure S2:** PCA loading plot combining grasses (red) and legumes (blue) demonstrating interspecific variation.

**a**


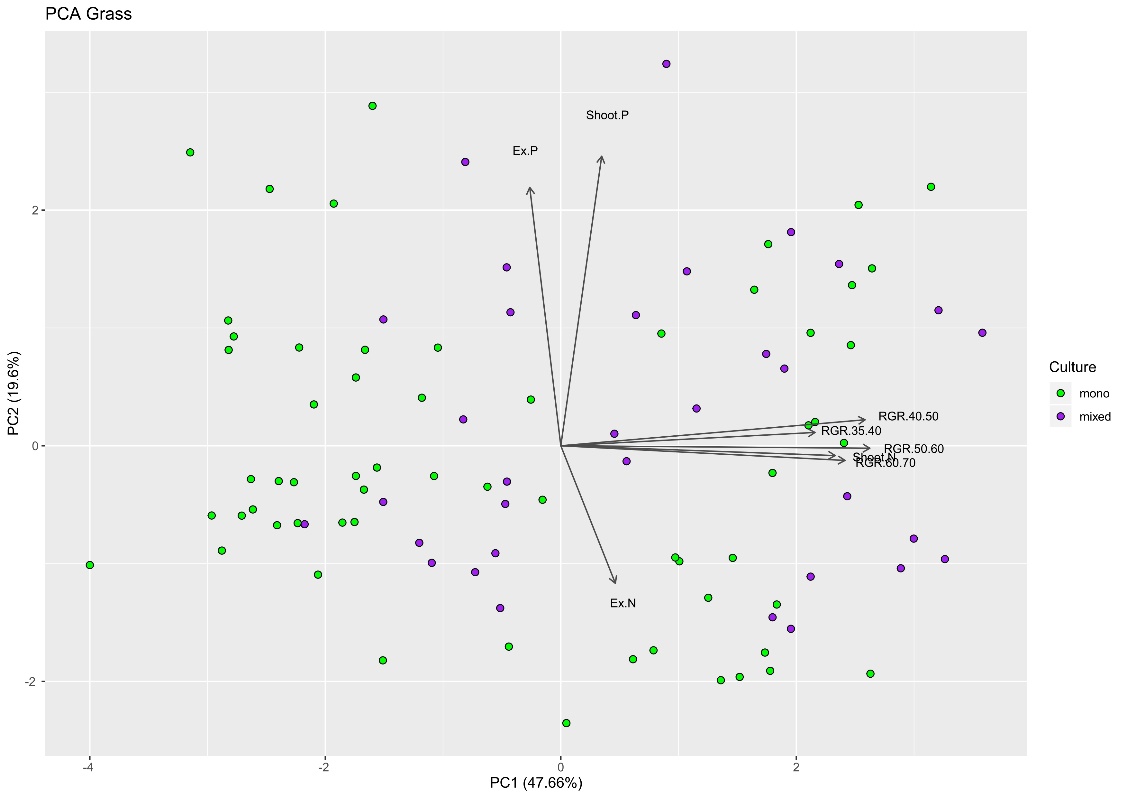


**b**

**Figure S3:** PCA loading plot for grasses (a) and legumes (b) demonstrating data spread between monocultures (green) and mixtures (purple).

**Table S1:** Varimax loadings for each principle components analysis. Significant factors were chosen based on eigenvalues larger than 1. Varimax values less than <0.30 were suppressed.

|  | Legume Monoculture | | | | Legume Mixture | | |
| --- | --- | --- | --- | --- | --- | --- | --- |
| **FACTOR** | 1 | 2 | 3 | 4 | 1 | 2 |  |
| **RGR 35-40** | -0.637 |  |  |  |  | -0.567 |  |
| **RGR 40-50** | -0.675 |  |  |  |  | -0.665 |  |
| **RGR 50-60** |  |  | -0.684 |  | -0.450 |  |  |
| **RGR 60-70** |  |  | -0.674 |  | -0.497 |  |  |
| **Shoot P** |  | -0.638 |  |  | -0.417 |  |  |
| **Shoot N** |  | -0.641 |  |  | -0.390 |  |  |
| **Ex P** |  | -0.372 |  | -0.491 | -0.393 |  |  |
| **Ex N** |  |  |  | -0.868 |  | -0.344 |  |
| **Eigenvalue** | 1.98 | 1.75 | 1.36 | 1.06 | 2.81 | 1.85 |  |
| **% Variance** | 24 | 21 | 17 | 13 | 35 | 23 |  |
|  | Grass Monoculture | | | | Grass Mixture | | |
| **FACTOR** | 1 | 2 |  |  | 1 | 2 | 3 |
| **RGR 35-40** | -0.395 |  |  |  | -0.393 |  |  |
| **RGR 40-50** | -0.470 |  |  |  | -0.489 |  |  |
| **RGR 50-60** | -0.482 |  |  |  | -0.461 |  |  |
| **RGR 60-70** | -0.431 |  |  |  | -0.424 |  |  |
| **Shoot P** |  | 0.685 |  |  |  | -0.666 |  |
| **Shoot N** | -0.423 |  |  |  | -0.454 |  |  |
| **Ex P** |  | 0.598 |  |  |  | -0.722 |  |
| **Ex N** |  | -0.368 |  |  |  |  | -0.923 |
| **Eigenvalue** | 3.96 | 1.66 |  |  | 3.59 | 1.5 | 1.06 |
| **% Variance** | 49.5 | 20.8 |  |  | 44 | 18 | 13 |
